# Prevention of ventilator-associated pneumonia in the surgical ICU at Siriraj Hospital

**DOI:** 10.1186/cc12436

**Published:** 2013-03-19

**Authors:** CH Pisitsak, O Chaiwat

**Affiliations:** 1 Siriraj Hospital, Bangkok, Thailand

## Introduction

VAP has continued to be a major cause of morbidity and mortality in critically ill patients in Thailand for decades. Previous research found that the implementation of VAP care bundles and the educational program can reduce VAP incidence in the ICU [1]. In this research we aimed to observe the reduction of VAP incidence after the implementation of VAP care bundles to ICU medical personnel.

## Methods

Inclusion criteria: all adult surgical patients (>18 years old) who are on ventilatory support in the surgical ICU at Siriraj Hospital. There are two groups, divided into pre-educational group (group I) and post-educational group (group II) (*n *= 220/group). We also observed the adherence rate to VAP care bundles according to the educational program. The pretest and post-test to determine the efficacy of the educational program were done. The VAP care bundles consisted of weaning according to weaning protocol, sedation vacation, head-of-bed elevation, measurement of cuff pressures four times/day, 2% chlorhexidine use for mouth care and emptying of ventilator circuit condensate.

## Results

There were 45.38 and 25.25 episodes of VAP per 1,000 ventilator-days in group I and group II, respectively (*P *= 0.020). The incidence of VAP was 21.82% in group I and 9.09% in group II (*P *= 0.000). There was significant reduction in the length of ventilatory support per person (group I = 2, group II = 1 (median), *P *= 0.013, 95% CI = 0.319 to 0.936) and mortality rate (group I = 15.5%, group II = 8.2%, *P *= 0.017). There was no significant difference in LOI, LOH and ATB cost. The pretest scores were 15.53 and 17.53 on average from 40 medical personnel in group I and group II, respectively (*P *= 0.000). The head-of-bed elevation adherence rate was improved after the educational program (group I = 50.1%, group II = 70.36%, *P *= 0.017). But the adherence to other bundles was not improved. See Tables 1 and 2.

**Table 1:** Impact of the educational program on outcomes

Outcome	Pre-education	Post-education	*P *value
VAP	45.38	25.25	0.020
Ventday	2	1	0.013
LOI	3	3	0.054
LOH	17	17	0.960
ATB cost	2,842.50	2,098.00	0.994
% death	15.5	8.2	0.017

**Table 2:** Adherence rate to VAP care bundles

Bundle	Pre-education	Post-education	*P *value
Wean	95.04	96.08	0.723
Sedate	92.26	95.28	0.467
HOB	50.10	70.36	0.017
Cuff pressure	87.29	80.78	0.278
M care	89.60	80.86	0.204
Drain	95.42	99.42	0.130

## Conclusion

The educational program and the implementation of VAP care bundles can reduce the incidence of VAP, length of ventilatory support and mortality rate in the ICU.
